# Perspectives on Remote Monitoring via Smartphones and Wearables Among Individuals With Lived Experience or at Risk of Eating Disorders (“This Could Go Very, Very Wrong”): Qualitative Interview Study

**DOI:** 10.2196/86382

**Published:** 2026-07-13

**Authors:** Carina Kuehne, See Heng Yim, Başak İnce, Elizabeth Fordham, Faith Matcham, Sara Simblett, Katie M White, Amos Folarin, Helen Sharpe, Ulrike Schmidt, EDIFY Consortium, Ulrike Schmidt

**Affiliations:** 1 Centre for Research in Eating And Weight Disorders, Institute of Psychiatry, Psychology and Neuroscience, King's College London, 16 De Crespigny Park, London, SE5 8AB, United Kingdom, 44 02080181; 2 Department of Psychology, University of Hong Kong, Hong Kong, China (Hong Kong); 3 Faculty of Science, Engineering and Medicine, School of Psychology, University of Sussex, Brighton, United Kingdom; 4 Department of Psychology, Institute of Psychiatry, Psychology and Neuroscience, King's College London, London, United Kingdom; 5 Department of Psychological Medicine, Institute of Psychiatry, Psychology and Neuroscience, King's College London, London, United Kingdom; 6 Department of Biostatistics & Health Informatics, Institute of Psychiatry, Psychology and Neuroscience, King's College London, London, United Kingdom; 7 Institute of Health Informatics, University College London, London, United Kingdom; 8 South London and Maudsley NHS Foundation Trust, London, United Kingdom; 9 NIHR Maudsley Biomedical Research Centre, London, United Kingdom; 10 School of Health in Social Science, University of Edinburgh, Edinburgh, United Kingdom; 11 See Acknowledgments

**Keywords:** eating disorders, mobile health, wearable devices, mobile phone, monitoring, qualitative

## Abstract

**Background:**

Remote measurement technology (RMT) is increasingly used in health research to collect real-world data relevant to clinical states (eg, sleep, activity, and stress). Concerns exist about the impact of remote tracking via personal devices and wearables on individuals with or at risk of eating disorders (EDs) by promoting a focus on exercise, diet, and appearance. There is a lack of research applying RMT to EDs.

**Objective:**

This study aimed to explore how smartphone- and wearable-based RMTs influence eating-, exercise-, and weight-related experiences among individuals with a history of or at risk of EDs and to identify perceived benefits, harms, and recommendations for their use in this population.

**Methods:**

In total, 14 semistructured interviews were conducted with former participants of Remote Assessment of Disease and Relapse: Major Depressive Disorder, a 2-year digital health study tracking depression outcomes via RMTs. Participants were included in this follow-up if they had disclosed a history of a comorbid ED or were within the at-risk age range (18-30 years) for EDs during Remote Assessment of Disease and Relapse: Major Depressive Disorder and displayed subclinical ED symptoms (Eating Disorder Diagnostic Scale). Interviews explored the impact of app engagement and wearables (Fitbits) on food, activity, and weight-related behaviors and attitudes. Template analysis was adopted to capture themes guided by the focus on ED-relevant domains.

**Results:**

In total, 6 themes captured participants’ experiences with RMTs across clinical status and presentation. Participants broadly appreciated the convenience and reflective potential, while some described emotional strain linked to constant self-tracking. Health data impacted participants’ eating and exercise habits through a dynamic process from awareness to cognition to action, fostering healthy routines or obsessive patterns, depending on emotional state, ED presentation, and recovery stage. Self-tracking appeared to mirror illness stage, supporting ED recovery among those with greater distance from illness, but risking reinforcement of compulsive patterns among those with residual or emerging symptoms. Participants’ recommendations for future studies in EDs stressed balancing autonomy with safeguards for vulnerable individuals.

**Conclusions:**

These exploratory findings, drawn from individuals with lived ED experience and young people at subclinical risk, suggest that RMT use was shaped by recovery stage and contextual factors, rather than being inherently beneficial or harmful. While findings should not be interpreted as evidence of RMT safety or acceptability in ED cohorts broadly, they raise important questions about ethical RMT design, including the selection of wearables, access to data, and researcher communication with participants.

## Introduction

Eating disorders (EDs) are serious mental illnesses, characterized by disturbed eating behaviors that typically emerge during adolescence or early adulthood, and carry among the highest mortality rates of all psychiatric conditions [1,2]. Despite significant advances in treatment, EDs still often follow a chronic course with low sustained recovery rates, highlighting gaps in understanding the dynamic factors that maintain them [3,4].

Recent years have seen a surge in the use of remote measurement technology (RMT) in health research, including wearable sensors and smartphone apps [5-8]. RMT facilitates ecologically valid self-reports and passive monitoring of behavior and physiology relevant to clinical states (eg, stress, sleep, and physical activity), offering promise for capturing the dynamic fluctuations that characterize conditions like EDs.

RMT can broadly be distinguished into research-grade devices (eg, Empatica and Ametris), which offer high data fidelity and greater researcher control over data feedback, and commercially available devices, such as consumer smartwatches (eg, Fitbit), which are more widely owned and embedded in daily life [9]. Research-grade devices tend to have lower acceptance rates due to their visibly clinical design, which may expose individuals as research participants [10,11]. Commercial devices carry social cachet, particularly among young people, making them attractive research tools and incentives for participation [12,13].

However, the features that make commercial RMTs appealing raise ethical concerns about the potential impact on vulnerable populations, particularly those with EDs. Studies suggest that technologies designed for behavior tracking can inadvertently exacerbate ED symptoms by promoting a focus on exercise, diet, and appearance in clinical and student populations [14-17], which may partly explain their limited application in ED research, despite increased use in other areas [18,19]. How these effects manifest may further depend on whether RMT is used self-guided or adjunctively alongside clinical care, where clinician involvement may buffer against harmful self-interpretations. Understanding how people with EDs and those with subclinical symptoms in emerging adulthood perceive and interact with these technologies within a research context is therefore critical to guide safe and appropriate future use.

To address this gap, we sought the perspectives from those with lived experience and those displaying subclinical symptoms as a first step before considering RMT implementation in more acutely ill populations. Specifically, we explored the experiences with commercially available RMT, a wearable wristband (Fitbit), and a smartphone app among individuals with a comorbid history of EDs and those within the critical age range of emerging adulthood. We drew on a subgroup of former participants from the Remote Assessment of Disease and Relapse: Major Depressive Disorder (RADAR-MDD) digital mental health study that remotely tracked clinical outcomes in people with depression from 2017 to 2020 [13,20]. Participation involved a 2-year-long assessment of mood, health, and behavior via smartphone sensors (noise, light, and location), “Fitbits” (heart rate and movement), and app-based tasks and questionnaires. Fitbits were provided for participants to use and keep as their personal devices, with data synced to university servers through pseudonymized study accounts. Participants attended an introductory session for guided device setup, but received no instructions on Fitbit use. Periodic check-in calls maintained engagement and addressed technical problems, but participants engaged with RMT in a self-guided manner, with no oversight and full access to their own data.

For this study, we reinvited former RADAR-MDD participants who had disclosed a diagnosis of a comorbid ED or were within the vulnerable age range (18‐30 years) at the time of participation and displayed subclinical ED symptoms at this follow-up. We aimed (1) to explore how participants perceived the influence of RMTs on their eating-, exercise-, and weight-related thoughts and behaviors; (2) to identify aspects of RMT experienced as helpful or harmful within this sample; and (3) to gather participants’ suggestions for designing future RMT research in dedicated ED cohorts.

## Methods

### Recruitment and Procedure

Potential participants were identified by RADAR-MDD researchers through screening of the original dataset, representing a convenience sample of those who consented to recontact. This approach was adopted as a follow-up to an established cohort with known RMT engagement to inform a future study of similar design in a dedicated ED cohort. No additional recruitment strategies were used.

Within this sample, inclusion criteria for this follow-up were designed to capture a broad range of ED-related experiences across clinical status and presentation [21,22]. Participants were included if they either (1) disclosed a history of an ED diagnosis during RADAR-MDD or (2) were aged 18‐30 years during RADAR-MDD and displayed subclinical ED symptoms at this follow-up. The age restriction was applied only to the subclinical group where age served as a proxy for developmental vulnerability, given evidence identifying emerging adulthood as the peak period for ED onset [23,24] and progression from subclinical to diagnosable conditions [25,26]. Individuals with a prior ED diagnosis were included regardless of current symptom status or age on the basis of their lived experience. Exclusion criteria included insufficient proficiency in English and lack of consent for the interview to be recorded. All participants had a history of major depressive disorder, as required for inclusion in RADAR-MDD (see Matcham et al [13] for the full list of inclusion criteria).

A total of 98 eligible participants were contacted via email (n=44 with ED history; n=54 meeting age criteria), with 1 follow-up reminder sent after 8 weeks. There were no active refusals. Interested participants completed the online consent form and screening survey, including demographic questions and the Eating Disorder Diagnostic Scale (EDDS) [27], a validated self-report measure to characterize current ED symptomatology [28,29]. EDDS responses were scored using established algorithms to derive diagnoses of anorexia nervosa (AN), bulimia nervosa (BN), and binge eating disorder (BED). Subthreshold presentations were classified where symptoms met criteria for Other Specified Feeding and Eating Disorders (eg, atypical AN and purging disorder) or indicated elevated risk below diagnostic threshold. The scoring algorithm is provided in Multimedia Appendix 1. For the subclinical group, EDDS scores indexed current risk. For participants with an ED history, scores were used descriptively rather than to determine eligibility to retain individuals with relevant lived experience whose symptoms fell below clinical cutoffs at the time of interview.

Upon survey completion, researchers arranged 1-hour online interviews via email.

### Data Collection

Interviews were conducted by trained researchers with backgrounds in psychology (CK and Bİ) between December 2022 and February 2023, video-recorded, and transcribed verbatim using Microsoft transcription software. Interviews were semistructured following a topic guide and codeveloped with clinicians and young people with lived experience from the youth advisory board of the EDIFY consortium [30] (topic guide provided in Multimedia Appendix 2). The transcriptions were checked for accuracy against the recordings and anonymized by CK and EF.

### Ethical Considerations

Ethics approval for the parent study RADAR-MDD was obtained from the Camberwell St Giles Research Ethics Committee in September 2017 (ref 17/LO/1154). Ethics approval for this follow-up was granted by the King’s College London Research Ethics Committee (HR/DP-22/23‐32898). For both studies, all participants provided written informed consent prior to study commencement. Interview attendance was reimbursed with a £20 Love2Shop voucher (GBP £1=US $1.32 as of June 24, 2026). Transcripts were anonymized, removing all identifiable information, and interview recordings were deleted within 6 months of study completion. Retained contact details (were consented to future contact) were not linked to the transcripts or the demographic information collected.

### Participants

In total, 15 (response rate 15.3%) participants aged 26‐66 years were recruited (female: n=13, male: n=1, and nonbinary: n=1). A total of 10 participants self-reported a history of an ED (mean age 48.2, SD 15.9 years), including AN (n=4), BED (n=3), BN (n=1), atypical AN (n=1), and avoidant or restrictive food intake disorder (ARFID; n=1). For most (n=9), EDDS scores indicated atypical AN presentations (ie, AN symptomatology without low body weight) or high risk for BN. Discrepancies between self-reported and EDDS-derived diagnoses likely reflect partial remission at the time of interview, differences in clinical and research diagnostic thresholds, as well as the time elapsed since formal clinical assessment given established diagnostic crossover between ED presentations [31-34].

Of the 5 participants without a diagnosed ED history (mean age 28.2, SD 1.7 years), 4 were classified as high risk based on EDDS responses suggestive of clinically relevant symptoms (eg, elevated weight and shape concern, dietary restraint, and low BMI) below full diagnostic threshold. One participant (P7) showed no indication of ED symptoms and was excluded from the final analysis. Participant information can be found in Table 1.

**Table 1.** Overview of participants in chronological order of interviews.

Participant ID	Gender	Ethnicity	Age^a^ (years)	Self-report diagnosis	Current EDDS^b^ classification	Education level	Employment status
P1	Female	White, Black African	65	BED^c^	Atypical AN^d^	Postgraduate degree	Student
P2	Female	White	59	BN^e^, BED	High-risk BN	Undergraduate degree	Student
P3	Female	White	66	ARFID^f^	Atypical AN	AS or A levels^g^	Retired due to illness
P4	Female	White	36	BED	Atypical AN	Undergraduate degree	Working
P5	Female	White	31	N/A^h^	High-risk poor body image	Undergraduate degree	Working
P6	Female	White	60	BED	Atypical AN	Undergraduate degree	Working
P7^i^	Male	Asian	28	N/A	No ED^j^	AS or A levels	Caregiver or parent
P8	Female	White	30	N/A	Atypical AN	Undergraduate degree	Working
P9	Female	White	31	Atypical AN	Atypical AN	Undergraduate degree	Working
P10	Female	White	29	AN	Atypical AN	Postgraduate degree	Working
P11	Nonbinary	White	26	AN	Atypical AN	Higher diploma	Not in education or employment
P12	Female	White	51	AN	Atypical AN	AS or A levels	Working
P13	Female	White	59	AN	No ED	Doctoral degree	Working
P14	Female	Black	26	N/A	High-risk poor body image	Vocational or professional qualification	Working
P15	Female	White	27	N/A	Atypical AN	Undergraduate degree	Working

a Age at time of participating in current interview.

b EDDS: Eating Disorder Diagnostic Scale.

c BED: binge eating disorder.

d AN: anorexia nervosa.

e BN: bulimia nervosa.

f ARFID: avoidant or restrictive food intake disorder.

g A-levels (advanced levels) are subject-based academic qualifications typically taken by students aged 16 to 18 years in the United Kingdom, and the primary entry requirement for university. AS levels (advanced subsidiary levels) are standalone 1-year academic qualifications in the United Kingdom, typically taken by 16- to 18-year-old students. They represent the first half of a full A-level.

h N/A: not applicable.

i Excluded from analyses.

j ED: eating disorder.

### Analysis

Template analysis was adopted using the method outlined by King [35], King [36], and Crabtree and Miller [37]. This approach was chosen for its flexibility in supporting both deductive analysis, guided by the study’s focus on emotional responses and behavioral changes related to eating, exercising, and body image, and inductive analysis grounded in participants’ lived experiences, capturing shared and distinct perspectives across age groups or ED presentations (eg, binge-type vs restrictive) [38,39].

Two authors (CK and SHY) independently reviewed 2 transcripts and generated initial codes informed by the interview guide. Codes were discussed and collaboratively organized into an initial hierarchical coding template of themes and subthemes. A further 2 transcripts were independently coded by both authors. Discrepancies and refinements to the coding template were discussed, before being applied to the remaining transcripts by CK, adding new codes as needed. SHY reviewed coding and interpretation of later transcripts to ensure richness and quality in theme development. The final template was reviewed jointly and imported into NVivo software (Lumivero) for data organization and analysis.

### Reflexivity

The lead author (CK) is a doctoral researcher in psychology with a professional interest in the use of digital tools in mental health research. CK, Bİ, and SHY work closely with individuals with EDs in research contexts, which informed sensitivity in conducting interviews and interpreting participants’ accounts. The research team included members with extensive clinical and research experience in EDs (US, Bİ, and SHY), which contributed to the interpretive depth and reflexive integrity of the analysis.

Analytic decisions were discussed regularly with coauthors to consider alternative interpretations and minimize bias. None of the researchers involved in data collection or analysis participated in the RADAR-MDD study, ensuring no prior relationship with participants. RADAR-MDD researchers (FM, SS, and KMW) contributed feedback on theme development, bringing firsthand knowledge of participants’ experiences that enriched interpretation.

## Results

### Overview

**Figure 1.**
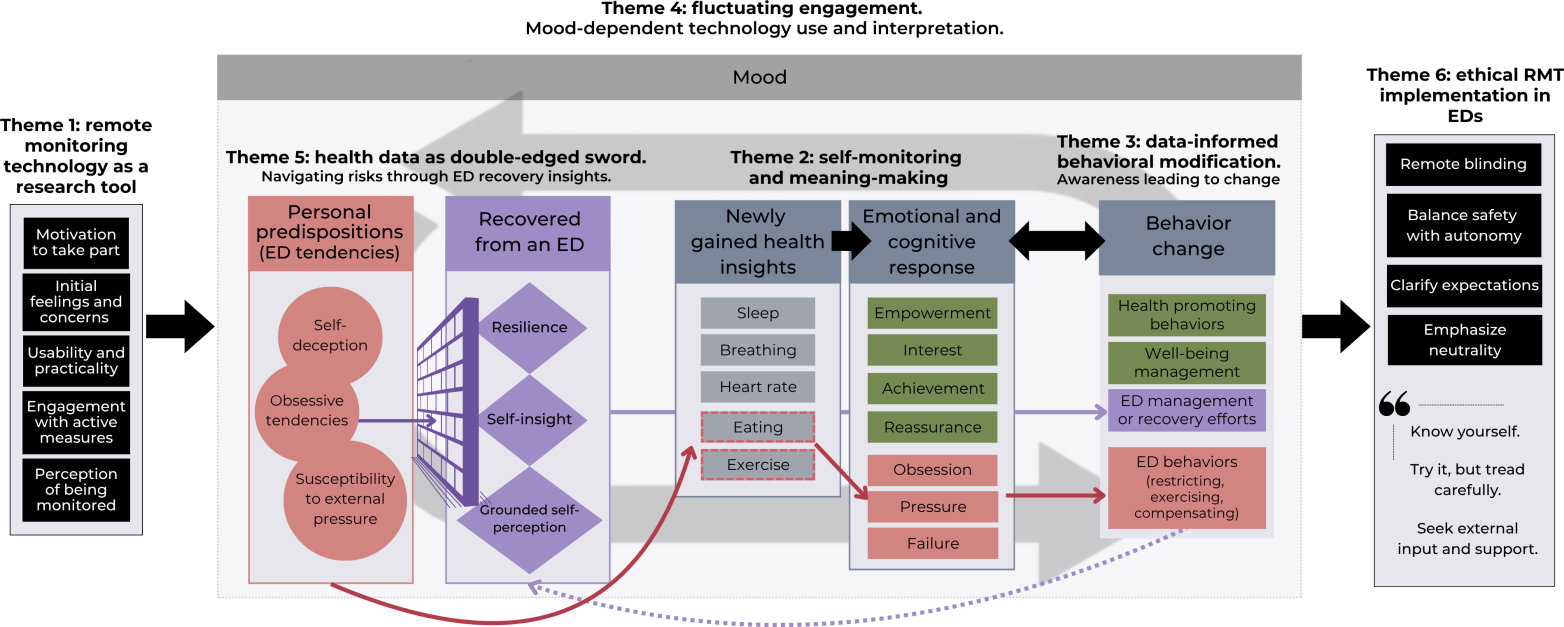
Visualization of the links between themes 1 and 6. Themes 2 to 5 form the central analytic arc, with colored arrows illustrating interconnections. Color use reflects conceptual functions; green reflects positive and red maladaptive processes. ED recovery (purple) can serve as a protective factor. Solid arrows reflect direct thematic progression, while dashed arrows represent reflective or indirect relationships. ED: eating disorder; RMT: remote measurement technology.

In total, 6 themes were generated (Figure 1). Theme 1 describes general experiences with RMT, serving as a contextual anchor, including participation motives and perceptions of being monitored. Themes 2‐5 form the core analytic arc, reflecting dynamic engagement with passive RMT (Fitbit) from awareness to cognition to behavior, shaped by mood. Theme 5 highlights the role of ED recovery in shaping whether self-tracking may be therapeutic or exacerbate ED tendencies. Theme 6 extends insights to future RMT design considerations in ED populations (codebook provided in Multimedia Appendix 3).

### Theme 1: RMT as a Research Tool: Motivations, Concerns, and Usability

This theme explores participants’ reflections on RMT as a general tool, covering usability, routine formation, and surveillance to provide context for the themes that follow.

#### Motivation to Take Part and Initial Feelings

Reasons to participate in RADAR-MDD mostly stemmed from personal relevance and altruistic motives, rooted in a desire to help others and belonging to something larger than oneself.

Many saw participation as an opportunity to gain insight into their own behaviors via RMT and described a perceived accountability by being monitored, hoping to support and manage their recovery.

I was looking for alternative routes for support because there was such a long waiting list at the eating disorder service. [...]. I just saw it as like, it’s an opportunity to give back, but also receive treatment at the same time, [...], it gave me a part to function and purpose to do things.

[Participant 11, ED history, AN]

The flexibility of remote involvement and minimal disruption made the study appealing to younger participants. The prospect of receiving a Fitbit was welcomed by both those intrigued by its novelty and those who found comfort in its familiarity. At the same time, there were concerns about its potential to reinforce compulsive behaviors among participants with a history of EDs, echoed by participants’ care teams.

I was fearful that if I started to collect lots of data, then I might get obsessive again about it, [...], you know, I feel guilty if I don’t get X amount of exercise done.

[Participant 13, ED history, AN]

There were mixed feelings from the eating disorder service when I actually started RADAR because they thought that the Fitbit would be ... used in a way for the eating disorder to, like carry on relapse symptoms.

[Participant 11, ED history, AN]

#### Usability and Practicality of RMT

Participants found the devices and apps user-friendly, with notifications to complete tasks supporting adherence. Some experienced technical difficulties or frustration with unpredictable scheduling of tasks; yet, most regarded them as an expected part of research and managed their time accordingly.

I spoke with my boss about it and just said like “Look, I’m taking part in this study. Occasionally, I might have to get my phone out to answer the questionnaires,” and she was okay with it. So in the end, it just became a lot easier to just answer them.

[Participant 8, subclinical risk of ED]

Participants described a similar adjustment period with the Fitbit where initial challenges, like discomfort or forgetting to charge or wear it, typically gave way to routine formation.

#### Cognitive and Emotional Engagement With Active Measures

Most participants appreciated the reflective opportunities facilitated by completing questionnaires, prompting moments of self-awareness, or encouraging the use of coping strategies to manage one’s mental health. Others found that the 2-week reporting intervals lacked nuance in capturing intense or fluctuating emotional experiences.

Some described how the constant emotion reporting could feel overwhelming or induce a sense of pressure around data consistency.

Some days when you felt actually okay, you felt a bit guilty for being okay. [...], I’m meant to be, like, giving them a good example [laughs]. But what if I had felt too good and what if it’s not the right data? So I worried a lot about it.

[Participant 12, ED history, AN]

#### Perception of Being Monitored

Participants were generally comfortable with being monitored remotely, expressing trust in the researchers and the anonymity of their data. Initial privacy concerns eased through clear information about data handling and study protocols.

Some participants reflected on generational differences in privacy concerns.

I think that my approach to it would definitely be a lot different compared to a younger person, [...] because I’m a bit older and more logical now. [...]. I definitely would have felt a bit tracked if I was a bit younger. But in my older years, I knew what I was doing.

[Participant 12, ED history, AN]

The research team’s communication over the study period was key to participants’ perception of being monitored. Positive interactions with researchers, regular study updates, and opportunities to connect with other participants added a human element to the study experience and a sense of connection. Some participants found comfort in being monitored, where the technology offered an emotional presence.

I think the other thing I found, sort of quite nice, [...], was that feeling that I wasn’t alone and that there was somebody sort of keeping an eye on me, which was quite comforting.

[Participant 2, ED history, BN and BED]

Others described a solitary engagement where the technology’s presence became internalized, taking the form of perceived expectations, leading to guilt around noncompletion.

It was quite an isolating experience. [...], it just felt like I had this little thing in my pocket, [...] or somebody on my shoulder going “right, you got to do this now,” “you got to do that now.” [...] I’d feel slightly guilty if I hadn’t gotten time to do a test. [...] like I’m not keeping my side of the bargain.

[Participant 12, ED history, AN]

### Theme 2: Self-Monitoring and Meaning-Making—Emotional and Cognitive Responses to Health Data

This theme describes participants’ experiences with the newfound health insights from the Fitbit, the emotional responses triggered by the information, and its impact on self-perception.

#### Gaining General Health Insights

As a widely shared experience, Fitbit data provided a newfound awareness of physiological patterns, such as sleep, breathing, and heart rate. These data were largely received with curiosity or enthusiasm, particularly when it helped contextualize day-to-day experiences.

I think that was good, [...], sort of seeing how I felt the next day depending on how much sleep I got and how good that sleep was.

[Participant 14, subclinical risk of ED]

The enthusiasm extended to visualizations to track progress and share quantifiable data with health care providers, providing a sense of validation and credibility of otherwise invisible conditions.

It gives me something to share with my GP as well. I can say, “Look, over the week, I haven’t slept very much, and I’ve been awake this amount of time.” [...] And having statistics helps your case, I think. [...] Because a lot of my health conditions, they’re not visible.

[Participant 1, ED history, BED]

#### Emotional Response to Activity-Related Data

Participants also reported increased awareness of their activity levels, facilitated by access to step counts, active minutes, or calorie expenditure. However, while general health data were widely appreciated, responses to activity-related data varied. For some, increased awareness and gamified elements such as streaks and milestone celebrations were experienced as rewarding and motivating, translating into a sense of agency to engage in physical activity.

It made me more mindful of when I wasn’t doing enough to do more, and I think that, that was good. It helped me, and I did improve. [...] to see which days I’m getting more steps than other days.

[Participant 1, ED history, BED]

In contrast, access to activity and performance-based metrics (eg, lung capacity) could also introduce stress or reinforce compulsive tendencies, fueled by standard fitness targets and accompanied by a sense of failure if unmet. This was observed in both restrictive- and binge or bulimic-type presentations.

I became quite obsessed about getting my 10,000 steps a day in. [...]. That became quite addictive for a while. [...], I became quite preoccupied with, you know, the details of how far I’ve walked and how many steps and how many calories it said I burnt.

[Participant 2, ED history, BN and BED]

When I first was wearing it, it was fine. And then after a while, I’d be in, like, step competitions [...]. Super, super unhelpful. [...], in that year I was diagnosed with atypical anorexia.

[Participant 9, ED history, atypical AN]

Increased awareness of calorie expenditure prompted some participants to reflect on their food choices, which could introduce a transactional mindset.

If I hit my step goal, I felt like I deserved a treat. [...], I felt a bit like a hamster in a bowl and just running for the treat, which was not great [laughs].

[Participant 8, subclinical risk of ED]

#### Impact on Self-Perception

For some, the impact of Fitbit data extended to body image and self-perception. Positive changes were more often described by older participants and those with binge-type presentations and attributed to increased physical activity and improved self-care. Notably, some participants’ accounts were tentative in how they characterized these positive experiences.

I think when I was using it in a, I say, in a positive way where I was using it to record my exercise, the knock-on effect was I generally tended to look after myself better and felt better, both physically and mentally.

[Participant 2, ED history, BN and BED]

External validation played a role among those for whom the visible presence of the Fitbit positively influenced their sense of self.

Wearing it made me feel ... better in a way about my weight and my body shape because it felt like I was doing something about it—[...], and that others judged me better than without it.

[Participant 4, ED history, BED]

Conversely, Fitbit’s focus on tracking weight could reinforce body image concerns, more commonly expressed by younger participants.

[I felt] more negative about myself. Because [...], on the home screen, it has your weight there. So if you are logging it regularly, you just have that number in your face. Which ... is not nice when you don’t like the number.

[Participant 8, subclinical risk of ED]

Among participants classified as high risk for a poor body image or low BMI, self-perception appeared largely unchanged, with Fitbit use not reported to influence how they felt about their bodies.

### Theme 3: Data-Informed Behavior Modification: Increased Awareness Leading to Change

This theme describes how emotional reactions to the data shaped behavioral change, including shifts in exercise routines, eating habits, and well-being management.

#### Influence on Exercising Behavior

The visibility of activity metrics prompted goal-oriented behaviors and motivated many participants to increase their physical activity. This effect mirrors the emotional responses described in the previous theme, with gamification features reinforcing both increased activity motivation and compulsive patterns to outperform previous goals.

It was kind of like a competition with yourself to try and do more steps than the day before. [...], I found it really motivating to have that on.

[Participant 4, ED history, BED]

And then you see [...]—“oh, I reached 10,000 steps” [...], and you’re like “OK, that’s good. Well, let’s see if I can do 15,000 or, you know, up to 20,000.”

[Participant 5, subclinical risk of ED]

Fitbit insights also prompted changes in the type of exercise undertaken.

I realised that cycling didn’t burn as many calories on the Fitbit as certain other activities. So I like, changed my exercise to ones that would, like, burn the most calories. [...], the metrics really changed what I would do and how I would do it.

[Participant 9, ED history, atypical AN]

While increased motivation for exercise appeared across participants, its manifestation differed between age groups. Older participants tended to portray their changed routines as shaped by gentle prompts, while younger individuals described a heightened sense of urgency.

Sometimes I might not be aware of how much I’m doing and if I’m not doing enough, [...], so I’ll try a bit harder tomorrow. [...]. It’s not a nagging voice [laughs], saying “You need to move more,” but it says it gently and it encourages you.

[Participant 1, ED history, BED]

I was like really, really trying to get in 10,000 steps no matter what. [...], like even if I was doing laps of the living room at the end of the night or whatever, just trying to get like the last few steps in [laughs].

[Participant 10, ED history, AN]

Notably, participants without an ED history, who were already active before the study, reported little to no influence from the Fitbit on their exercise habits.

#### Influence on Eating Choices

While exercise-related behaviors were more directly influenced by the Fitbit, dietary choices remained largely unaffected, with many participants being unaware of the app’s food tracking features.

Behavioral changes occurred more often among younger participants, who described adjustments to their food intake based on energy expenditure.

I would eat something sweet, [...] because I’d done 10,000 steps, so I do deserve it [laughs]. [...]. I probably wouldn’t give myself any treats if I hadn’t hit my step goal.

[Participant 8, subclinical risk of ED]

For others, the health-focused mindset fostered by tracking exercise acted as a trigger for behavior change, though this was often voiced tentatively, reflecting uncertainty about the nature of the change.

I may have been trying to eat more healthfully because I was on a bit of a health trip because I was exercising and recording my, tracking my exercise and so on, [...], as a sort of side effect of that.

[Participant 2, ED history, BN and BED]

#### Well-Being Management

Health insights were also applied to managing emotional well-being, with tracking features offering a tangible indicator for recognizing and regulating stress.

It monitors my pulse, [...] I was having panic attacks last year and found that my pulse would be racing. And so if I see that now, I know how to calm myself down [...], I find that quite a helpful tool.

[Participant 4, ED history, BED]

### Theme 4: Fluctuating Engagement: Mood-Dependent Technology Use and Interpretation

The interviews revealed a dynamic, bidirectional relationship between participants’ emotional states and technology engagement. Initial periods of intense engagement linked to its novelty often gave way to habituation. Engagement fluctuated with mood, often rising with higher energy and declining during low mood, either from disinterest or as a protective strategy. Conversely, mood could shape the interpretation of the same data.

If I was in the right headspace, yeah, then it would be [empowering]. [...], if my mood was bad, it would just make me feel bad.

[Participant 1, ED history, BED]

These patterns emerged across the sample, but reflections among younger participants with restrictive ED histories often revealed a more complex emotional journey of cycles of tracking and reactive disengagement.

It would be like a month at a time that I’d be, like, really obsessed with that thing. And then I’d be like “OK, now I have to stop looking at it.” I had to, [...] like, stop myself from there.

[Participant 10, ED history, AN]

Participants with binge-type presentations and more established recovery insight more often described proactive disengagement to avoid feelings of inadequacy.

Sometimes I could look at it and it would encourage me. And other times I would try and avoid it. [...]. If I’m at the bottom of a bad spiral, nothing is going to help, so ... I pretty much hibernate [laughs], you know, and sort of avoid everything until I come back up again.

[Participant 1, ED history, BED]

### Theme 5: Health Data as a Double-Edged Sword: Navigating Risks Through Recovery Insights

This theme describes participants’ reflections on how their personal vulnerabilities and insights gained from ED recovery shaped the line between helpful self-monitoring and harmful tracking.

#### Recognizing the Risk

Across participants, activity tracking was widely recognized as risky in the context of EDs. Drawing on their personal experience or observations of close others, fitness trackers and their seductive potential of self-quantification were acknowledged as tools that could easily be used to encourage or justify disordered self-discipline patterns.

Because I have worn fitness devices and things in the past, I do know that [...] it can have an effect on me because it can obviously make me more aware of my levels of physical activity. [...], that was just something that I had to be very aware of.

[Participant 10, ED history, AN]

Reflections on past ED behaviors also revealed a self-deceptive nature of the disorder.

I keep thinking back to younger me and how self-deluded I often was. [...] I can imagine I would have gone, “Oh yes, I’ll be good and I’ll do this,” but then, [...] I would definitely want to find a way to switch it [calorie tracking] back on.

[Participant 2, ED history, BN and BED]

#### Shifting Perspectives Through Recovery

Recognizing the risks of activity tracking was facilitated by increased self-insight, reduced reliance on numerical feedback, and more grounded self-perception that participants attributed to having recovered from their ED.

I don’t have an eating disorder anymore. [...]. I’m aware of my body size as it is and my ideals and my body shape without having to look at a wearable to know about it.

[Participant 3, ED history, ARFID]

For some, this also involved developing an understanding of the wider influences on disordered eating, as well as their own needs and boundaries.

There’s other factors that come into play, like peer pressure, social media pressure, [...]. I’ve learned so much about how the body chemistry works around food. [...] Knowledge is power, but it’s hard to get that understanding when the food industry in our society is so powerful.

[Participant 6, ED history, BED]

#### Gained Self-Insights Applied to Data Engagement

With increased distance from active illness and a developed awareness of personal vulnerabilities, participants described approaching certain features cautiously or disengaging intentionally.

I think I used it [food tracking] once and then I was like “Actually there’s probably a way, that this could go very, very wrong.”

[Participant 11, ED history, AN]

Self-regulation could also translate into reclaiming choice, engaging with the technology in ways that aligned with personal needs and skills without feeling compelled by the data.

It’s easy to get quite fixated on how many steps you’ve done a day, you know. But I set myself a sensible target and tried to do that.

[Participant 3, ED history, ARFID]

Some participants framed their engagement with tracking data positively, attributing this to emotional distance from their ED, which buffered against unhealthy patterns.

It was helpful, it was a motivator. [...] because, I mean I don’t have an eating disorder anymore and I’m just not that obsessive in the way that I used to be. [...], it doesn’t take over in the way it used to.

[Participant 2, ED history, BN and BED]

However, participants also acknowledged the fragility of recovery, where awareness of risk did not necessarily prevent engagement with tracking data.

I think you have to have quite a lot of, a strong will. Especially if you’re in recovery. If you’re just having, like a bad day, you could just be like “oh just I’ll just turn it back on for the day.”

[Participant 9, ED history, atypical AN]

#### Data Used for ED Management and Fitbit as a Therapeutic Tool

When approached with insight and intentionality, the Fitbit could become an active part of ED management, offering positive reinforcement for recovery efforts and the ability to engage on one’s own terms.

I think with a Fitbit, it’s a friend on your wrist who’s there when you need them. [...]. And it’s not judgmental, [...]. Because most people with eating disorders already know that there’s something up [...]. And the last thing they need is somebody nagging them or making them feel bad. [...]. But interacting with an app, [...], it’s a little easier.

[Participant 1, ED history, BED]

Objective data grounded distorted self-perceptions in observable reality and supported self-control and behavioral change.

There were times when I was really sure that I’d gained loads and loads of weight, [...], but then I could go back and look and actually, [...] my weight was quite stable, [...]. It was just reassuring.

[Participant 10, ED history, AN]

Technology also served as a more validating alternative to traditional ED services for those who felt let down by standard care.

It was a productive and more purposeful way of treating an eating disorder than going to the eating disorder service and being patronised or gaslit. [...]. I could limit my steps and stuff, [...], so I wasn’t over-exercising. [...], it was an effective form of treatment at the time.

[Participant 11, ED history, AN]

Notably, this reliance on self-monitoring became a double-edged sword, with excessive dependence leading to distress when the device was unavailable.

It was such an effective piece of equipment that I relied on for routine. When it broke, I felt lost, and that’s when I relapsed.

[Participant 11, ED history, AN]

Others saw engaging with potentially triggering technology, like activity trackers, as a manageable challenge that can support growth, provided it is approached with appropriate boundaries and external support.

### Theme 6: Participant Recommendations for Ethical RMT Implementation in EDs

This theme describes participants’ reflections on the potential benefits and risks of RMT studies for young individuals with EDs, drawing on their own experience of taking part.

#### Advice for Prospective Young Study Participants With EDs

Participation was widely framed as a valuable opportunity for personal insight and self-management facilitated by self-monitoring and for the potential to support others.

I would probably recommend to give it a go. [...]. I think it’s a prime example of prevention in the community. [...]. I think Fitbits could actually save lives, in terms of like eating disorders.

[Participant 11, ED history, AN]

At the same time, there was a shared emphasis on the importance of self-reflection and external perspectives from friends, family, or health care professionals in assessing whether participation would be constructive or risky alongside transparency about the research’s purpose and risks.

[I’d say], think about how it might feel to do this over time. It might not be something for everybody who’s going through extreme anorexia [...], so maybe make sure that a doctor is okay with [you] doing it.

[Participant 12, ED history, AN]

Some participants expressed more definitive views against the use of activity trackers during acute illness, regardless of the context.

If I was being completely honest, I would be like “Do not wear an activity tracker if you have an active eating disorder, just don’t do it. It’s just not worth it.”

[Participant 9, ED history, atypical AN]

#### Design Considerations for Future Studies

Several recommendations were offered to enhance the safety of future RMT studies involving young people with EDs in earlier stages of illness or recovery. A central concern was balancing autonomy with protection. Given the self-deceptive nature of EDs and the seductive pull of self-quantification, some saw managing access to metrics as a key safety consideration, while others advocated for flexible engagement on their own terms to avoid patronizing experiences.

I think removing that kind of choice is important. [...], managing the metrics or what they can and can’t see I think is really important.

[Participant 9, ED history, atypical AN]

You don’t really want to take autonomy away from anyone, you know. It’s really difficult.

[Participant 8, subclinical risk for ED]

Transparency around study purpose and expectations was seen as crucial for safe engagement and avoiding feelings of surveillance or guilt.

I never really knew whether it was OK if I didn’t take part in that day. [...]. And if somebody said, you know what, you don’t actually need to do every single day as long as you do this amount percentage of the time. Then that would be great.

[Participant 12, ED history, AN]

Clear communication about researcher neutrality and the noncomparative use of data was seen as vital to prevent negative self-evaluation.

And just being like “Hey, [...],we don’t care about how many steps you’re doing. Like, we’re not measuring that out of judging you or saying that’s enough. We just need that information.”

[Participant 9, ED history, atypical AN]

A final consideration was the impact of age on engagement with RMT, with some participants reflecting on how their younger selves might have struggled with self-monitoring in ways that they were now more equipped to navigate. They suggested that younger participants may require additional guidance to manage expectations, stress, or guilt associated with study participation.

## Discussion

### Principal Findings

This study explored how individuals with a history of EDs and young people at high risk for ED symptoms experienced remote monitoring via wearables and smartphones as part of a longitudinal mental health study. Reflecting the perspectives of those with partially or fully remitted symptoms, alongside a subclinical at-risk group, findings suggest that tracking technology in this sample was neither uniformly beneficial nor harmful but shaped by individual vulnerabilities and contextual factors. While these insights are exploratory, they seem to align with the mixed literature on fitness tracking in nonclinical populations [17,40-43] and raise important questions for the ethical implementation of RMT in ED research.

### Recovery Status as a Lens of Interpretation

Participants’ experiences centered on the double-edged nature of empowerment and distress associated with self-monitoring and health-related metrics. The emotional and behavioral impact of self-tracking was often not determined by the data itself, but mediated by individuals’ psychological positioning, ED histories, and level of recovery insight. Echoing existing concerns, self-tracking’s emphasis on quantification and gamification could introduce or reactivate obsessive mindsets and compulsive behaviors [44,45], a risk participants reflected may be heightened during periods of active illness. Yet, with greater distance from an active ED, participants’ perspectives described here suggest that the same tools may offer reassurance and behavioral regulation by validating positive change and helping manage emotional states.

Self-tracking may mirror individuals’ current relationship with their bodies and sense of control, depending on the degree of self-awareness or rigidity with which it is approached. This differentiation suggests that recovery is not only a marker of clinical change but also an interpretive lens through which technology use is navigated. Yet, this capacity may be fragile, as the allure of data could override recognition of harm and trigger relapse risk. Additionally, the tendency of EDs to obscure recognition of harm [46,47] means that accounts described as positive and health-promoting can coexist or overlap with compulsive behaviors. Indeed, the tentative framing with which some participants voiced positive experiences may signal ambivalence and represents a caveat for interpreting the findings above.

Overall, this interpretive variability seems to support the need to move beyond fixed assumptions about the effects of digital tools. As recovery from an ED involves a shift away from rigidity and external validation [48-50], so too may the relationship with tracking technologies, functioning as another challenge to be navigated. Contrary to the assumption of some binge-type ED intervention models that use of a tracking tool in itself drives positive behavior change [51,52], these findings would suggest that without recovery insight, tracking may worsen behaviors. Notably, these observations were grounded in participants’ retrospective reflections on earlier illness stages and warrant prospective investigation among participants in more acute stages of illness.

### Self-Monitoring as Relational Agent

Technology-mediated monitoring could be experienced as an external, reassuring presence (“someone is checking in on me”) or as a solitary, watchful presence (“someone on my shoulder”). Participants often anthropomorphized app assessments and their Fitbit, describing them as a “friend on their wrist” or “nagging voice.” This highlights that while passive sensing tools collect data objectively, the meanings users assign to that data are subjective and relational [53], a consideration that deserves attention in how RMTs are deployed in research and clinical contexts.

This relational dynamic also surfaced in the tension between RMT experienced as a substitute for traditional support where conventional ED services had failed and RMT described as safe only with clinical oversight, particularly during earlier illness stages. The relational interpretation of digital tools, including computerized ED self-help, has previously been noted [54] and may be particularly salient in young ED populations, where self-objectification and surveillance are already core pathology features [55-57]. This suggests that rather than positioning RMT as adjunct or alternative to clinical care, the relationship participants form with the technology may shift depending on recovery stage and prior experience with or access to care. Designers of RMT studies and technologies should thus consider not only what is being measured but also what relational meanings they evoke.

### Repositioning RMT Engagement as Situated and Temporally Fluid

Participants described often cyclical engagement with RMTs, mirroring fluctuations in mood and mental health. This pattern is commonly observed in digital research in populations managing chronic or episodic conditions [58-60] and complicates assumptions about participant engagement as a static metric of user adherence or technological acceptability [61]. Instead, some participants described a deliberate boundary setting to manage well-being. Mood not only shaped willingness to engage but also was itself influenced by data, reinforcing the cyclical nature of this dynamic.

Informed by participants’ retrospective accounts of their own more acute periods, participants with EDs in RMT studies may thus face the added burden of regulating their engagement with digital tools that could exacerbate distress. This raises questions about the suitability of interactive data collection methods in populations at earlier stages of illness, where disengagement may be a protective response rather than a sign of nonadherence.

### Balancing Autonomy and Protection in RMT Use

The ambivalence in participants’ experiences surfaces as the ethical tension between autonomy and protection in RMT use in ED populations. In the context of EDs, where experiences of control, autonomy, and moral self-evaluation are particularly salient [62,63], removing choice might risk alienating participants, while unrestricted access may expose them to preventable distress. Although agency and choice were perceived as critical, this was not absolute: participants viewed data obscuring as protective, provided it was justified, transparently explained, and implemented respectfully. Participants recommended that this should be accompanied by honest reflection and third-party perspectives in assessing readiness to participate. This aligns with recommendations for enhanced informed consent in digital health research, including prescreening for vulnerability, collaborative decision-making, and ongoing consent [64].

Within this self-selected sample of individuals with established research engagement, remote monitoring and data privacy were largely met with nonchalance, which participants attributed to transparent communication and the approachability of the research team. This suggests that trust in institutions and relationships with researchers play critical roles in comfort with monitoring, more so than the nature of the data collected itself, and may be particularly applicable to ED populations prone to guilt and shame [65,66].

### Implications for RMT in ED Research and Clinical Practice

The following implications are grounded in the experiences and recommendations of a predominantly recovered sample and younger people at subclinical risk and are offered as directions for future inquiry and design, rather than as implementation evidence. Within these bounds, findings point toward the need for flexible, user-centered approaches to RMT design, which may be especially critical in ED populations. If recovery status shapes how individuals interpret and respond to health data, it is essential to support users in navigating that relationship. Tailoring RMTs to presentation (eg, restrictive vs binge- or bulimic-type), age, and recovery stage may help mitigate harm and enhance engagement.

Tailoring could include selecting purely passive sensors over interactive devices and adjusting access to metrics (eg, blinding and opt-in to feedback) to protect vulnerable groups and avoid confounding behavior change, as implemented in recent RMT studies [67-70]. Further research is needed to examine how these dynamics operate in individuals experiencing acute or severe ED symptoms.

Of note, while Fitbit use in RADAR-MDD allowed engagement and functioned as an incentive for taking part, most participants reported approaching the device as a means of contributing data, rather than altering behavior. Passive wearables may thus be more appropriately framed solely as research instruments, with clear boundaries around access and use. Research teams should consider co-design approaches with individuals with lived ED experience in shaping study protocols and use transparent, empathic communication. Future RMT implementations would also benefit from incorporating clinician expertise in ED psychopathology, both in study design and in supporting participants’ engagement with self-tracking data.

Finally, clinical services may benefit from integrating RMTs into care. Given that participants assigned relational meanings to RMTs, these tools may be most effective when embedded in supportive therapeutic relationships that help contextualize the data. However, our findings indicate that their utility may depend on stage of recovery and prior experiences with care, suggesting that uniform approaches to implementation may not be suitable.

### Limitations

First, the demographic homogeneity and inclusion of clinical and subclinical participants across presentations, while intentional for this exploratory study, limit how broadly findings can be interpreted. Many participants with an ED history showed partial or full remission at the time of interview, and at-risk participants may not transition to a diagnosable ED. Reflections may differ for individuals who are in earlier or more acute stages of their ED, and potential harms or benefits of RMT observed here should be understood as exploratory.

Second, the sample was self-selected and drawn from a single pre-existing cohort, limiting the diversity of perspectives captured. Some individuals with an ED diagnosis declined participation in the original RADAR-MDD study due to concerns about symptom exacerbation [71], potentially resulting in underrepresentation of those most vulnerable to harm. All participants carried a long-standing history of major depressive disorder, a condition associated with sustained self-management and help-seeking behaviors [72,73]. This overlap may reflect a greater degree of self-awareness than among participants with noncomorbid ED. The reliance on a comorbid cohort reflected a pragmatic necessity as, to our knowledge, no study of comparable design existed in a dedicated ED cohort at the time of this study. Future studies should use purposive sampling strategies to ensure representation across ED presentations, illness severity, demographic characteristics, and monitoring devices.

Finally, Fitbit-based monitoring allowed engagement with real-time feedback, meaning participants’ experiences may have largely reflected on using a commercially available tracker within a research context, and findings may not generalize to other forms of “truly” passive sensing. Importantly, participants acknowledged that they would not have used a Fitbit outside the context of study participation and often transitioned away from wearing the Fitbit after the study concluded, lending weight to the relevance of the findings. Future research should compare commercially available and research-grade devices to better understand how design and data visibility influence acceptability and risk in ED populations.

Despite these limitations, the study makes an important contribution to a rarely explored intersection between RMT use and ED experiences, including individuals with lived experience and at high risk, across age groups and presentations. The clinical ED expertise within the research team represented an interpretive strength, particularly in contextualizing ambivalent accounts. Reporting was conducted in accordance with the COREQ (Consolidated Criteria for Reporting Qualitative Research) guidelines (Checklist 1).

### Conclusions

RMTs hold promise for understanding and supporting individuals with EDs, offering unique insights into behaviors, physiology, and mood in daily life. Yet, they carry substantial risks if implemented without sensitivity to the complex dynamics of self-monitoring in ED recovery. Grounded in the reflections of individuals with lived ED experience and younger people at subclinical risk, this study suggests that the impact of RMTs seems to be shaped by the user’s ED history and recovery insight, rather than by the technology itself. Participants’ insights raise important questions for researchers and clinicians to consider and offer a foundation for future work aimed at refining ethical and user-centered approaches to RMT implementation in ED populations.

## Supplementary material

10.2196/86382 Multimedia Appendix 1 Eating Disorder Diagnostic Scale scoring.

10.2196/86382 Multimedia Appendix 2 Interview topic guide.

10.2196/86382 Multimedia Appendix 3 Final codebook.

10.2196/86382 Checklist 1 COREQ checklist.

## Abbreviations

AN: anorexia nervosa
ARFID: avoidant or restrictive food intake disorder
BED: binge eating disorder
BN: bulimia nervosa
COREQ: Consolidated Criteria for Reporting Qualitative Research
ED: eating disorder
EDDS: Eating Disorder Diagnostic Scale
RADAR-MDD: Remote Assessment of Disease and Relapse: Major Depressive Disorder
RMT: remote measurement technology
