# Supplementary material for: Perspectives on Remote Monitoring via Smartphones and Wearables Among Individuals With Lived Experience or at Risk of Eating Disorders (“This Could Go Very, Very Wrong”): Qualitative Interview Study
Source: JMIR Form Res. 2026 Jul 13;10:e86382. doi: 10.2196/86382 (PMC13362873; doi:10.2196/86382)
Supplement: Multimedia Appendix 1 [file formative-v10-e86382-s001.docx]

* Encoding: UTF-8.

**EDDS SCORING FOR BASELINE** .

*Create GENDER numeric variable for EDDS scoring. ‘Other’ and ‘Prefer not to say’ will be included as female (more stringent cut-offs).

COMPUTE edds_gender=999.

If gender=1 edds_gender=1.

If (gender=2 or gender=3 or gender=4 or gender=5) edds_gender=2.

RECODE edds_gender (999=SYSMIS).

*To adjust binge eating frequency in case of errors in recording.

If edds4=0 edds5=0.

If edds4=0 edds6=0.

If edds4=0 edds7=0.

If edds4=0 edds8=0.

If edds4=0 edds9=0.

If edds4=0 edds10=0.

If edds4=0 edds11=0.

If edds4=0 edds12=0.

EXECUTE .

*Correct for when binge eating is reporting without loss of control .

If (edds4=1 and edds5=0) edds6=0 .

EXECUTE .

*For Diagnosis.

COMPUTE feature=sum.1(edds7,edds8,edds9,edds10,edds11).

COMPUTE binge=999.

If (edds4=0 and edds5=0 and edds6<2) binge=0.

If (edds4=1 and edds5=1 and edds6>=2) binge=1.

If (edds4=1 and edds5=1 and edds6>=4) binge=2.

RECODE binge (999=SYSMIS).

EXECUTE .

COMPUTE compsum=999.

COMPUTE compsum=SUM.2(edds13, edds14, edds15, edds16).

RECODE compsum (999=SYSMIS).

COMPUTE compen=999.

If compsum<=1 compen=0.

If compsum>=2 compen=1.

If compsum>=4 compen=2.

RECODE compen (999=SYSMIS).

EXECUTE .

COMPUTE purge=999.

COMPUTE purge=SUM(edds13, edds14).

EXECUTE .

COMPUTE wtshap=999.

If edds3<2 wtshap=0.

If edds3>=2 wtshap=1.

If edds3>=4 wtshap=2.

EXECUTE .

COMPUTE lowBMI=999.

If bmi>10 lowBMI=0.

If (age_check>=18 and bmi<=18.5) lowBMI=1.

If (edds_gender=1 and age_check=17 and bmi<=18) lowBMI=1.

If (edds_gender=0 and age_check=17 and bmi<=18.5) lowBMI=1.

If (edds_gender=1 and age_check=16 and bmi<=17.75) lowBMI=1.

If (edds_gender=0 and age_check=16 and bmi<=18) lowBMI=1.

EXECUTE .

COMPUTE fearwt=999.

If edds2<6 fearwt=0.

If edds2>=2 fearwt=1.

If edds2>=4 fearwt=2.

RECODE purge wtshap lowBMI fearwt (999=SYSMIS).

EXECUTE .

COMPUTE wtloss=999.

If (weight>(0.9*(heighest_weight))) wtloss=0.

If (weight<=(0.9*(heighest_weight))) wtloss=1.

RECODE wtloss (999=SYSMIS).

EXECUTE .

*Diagnostic groupings .

COMPUTE eddsdx=999.

EXECUTE .

*AN .

If (lowBMI=1 and fearwt=2 and wtshap=2) eddsdx=1.

If (lowBMI=1 and compen=2 and wtshap=2) eddsdx=1.

EXECUTE .

* Atypical AN .

If (wtloss=1 and fearwt=2 and wtshap=2 and eddsdx=999) eddsdx=4.

If (wtloss=1 and compen=2 and wtshap=2 and eddsdx=999) eddsdx=4.

EXECUTE .

*BN .

If (binge=2 and compen=2 and wtshap=2 and eddsdx=999) eddsdx=2.

EXECUTE .

*Low freq BN/ BN type-presentation (includes lower threshold for BI concerns).

If (binge=1 and compen=1 and wtshap=1 and eddsdx=999) eddsdx=5.

If (binge=1 and compen=1 and wtshap=2 and eddsdx=999) eddsdx=5.

If (binge=1 and compen=2 and wtshap=2 and eddsdx=999) eddsdx=5.

If (binge=2 and compen=1 and wtshap=2 and eddsdx=999) eddsdx=5.

If (binge=2 and compen=2 and wtshap=1 and eddsdx=999) eddsdx=5.

If (binge=2 and compen=1 and wtshap=1 and eddsdx=999) eddsdx=5.

EXECUTE .

* BED .

If (binge=2 and feature>=3 and edds12=1 and compen=0 and eddsdx=999) eddsdx=3.

EXECUTE .

*Low freq BED .

If (binge=1 and feature>=3 and edds12=1 and compen=0 and eddsdx=999) eddsdx=6.

EXECUTE .

*Purging disorder .

If (purge>=4 and binge=0 and wtshap=2 and eddsdx =999) eddsdx=7.

EXECUTE .

* Night eating syndrome .

If (edds17>=4 and eddsdx =999) eddsdx=8.

EXECUTE .

* High risk bulimic-type (binging, purging or compensatory behavious) .

If ((binge>=1 or compen>=1) and eddsdx = 999) eddsdx = 9 .

EXECUTE .

* High risk weight and shape concerns / low BMI.

If ((wtshap>=1 or fearwt >= 1) and eddsdx = 999) eddsdx=10 .

If ((lowBMI=1) and eddsdx=999) eddsdx = 10 .

EXECUTE .

*all else no ED .

RECODE eddsdx (999=0) .

EXECUTE .

*Add value labels to eddsDX variable.

VALUE LABELS eddsdx

'0' 'No eating disorder / low risk'

'1' 'Anorexia nervosa'

'2' 'Bulimia nervosa'

'3' 'Binge eating disorder'

'4' 'Atypical anorexia nervosa'

'5' 'Low frequency bulimia nervosa / BN-type presentation'

'6' 'Low frequency binge eating disorder'

'7' 'Purging disorder'

'8' 'Night eating syndrome'

'9' 'High risk bulimic-type'

'10' 'High risk poor body image or low BMI' .

EXECUTE.

VARIABLE LABELS eddsdx 'ED Type' .
