# Supplementary material for: Perspectives on Remote Monitoring via Smartphones and Wearables Among Individuals With Lived Experience or at Risk of Eating Disorders (“This Could Go Very, Very Wrong”): Qualitative Interview Study
Source: JMIR Form Res. 2026 Jul 13;10:e86382. doi: 10.2196/86382 (PMC13362873; doi:10.2196/86382)
Supplement: Multimedia Appendix 2 [file formative-v10-e86382-s002.docx]

**Introduction**

Welcome! Thank you for making the time. I am [name] and I’m part of the ED Research Team at KCL. I will be doing the interview with you today. I can see that you have completed the consent form, thank you.

**Explain purpose & procedure of interview**

Before we start with the interview, I am going to give you a recap on the study information. This is part of the protocol, so please bear with me and do let me know if you have any questions.

We are planning a research study, which will focus on the recovery journey of young people with eating disorders. And like the RADAR study, in which you have previously participated, we plan to use smartphones and wearable trackers, such as Fitbits. Our goal is to understand how people recover from eating disorders, and what factors make this harder or easier, quicker or slower. Using this information, we hope to provide a ‘map’ for clinicians to help them tailor treatments to a person’s needs.

However, before we start with our planned study, we would first like to hear from people like you, who already have experience with these devices. In today’s interview, I will focus on how participating in the RADAR study might have affected your thoughts or behaviours surrounding your physical activity, your eating and body image. Please be as open as you feel able to about your experiences, we are interested in positive and negative aspects, as this will help us understand how to best design our new study. You don’t have to answer any questions you don’t feel comfortable answering. You might have already completed an interview for the RADAR study, and while some questions might be similar to what you have previously discussed, our main focus here is quite different. We also don’t have access to any earlier interviews, so please do provide us with as much detail as possible.

This interview will take about one hour and will be recorded, so that I don’t have to take as many notes and can give full attention to your answers. Please let me know if you would like to pause the recording at any point. After the interview, I will type up the recording and delete the recording file. The write-up from our conversation will be anonymous and not include any information that could identify you, such as names or locations. If you change your mind about participating after the interview, you will have up to 5 working days to completely withdraw from the study and in that case, we will not include your interview. Please note that after 5 working days, it will not be possible to withdraw your interview, because by then we will have started to analyse it. Once we have conducted all the interviews, we will write a report that might be published in a journal. This report may include some quotes from you as examples, but these will be anonymous and not connected to you in any way.

Does that sound okay? Do you have any questions before we start?”

***Start recording***

**Opening questions: General experiences about participation in RADAR-MDD**

1. To begin with I would like to ask you to cast your mind back to the start of the RADAR study. Do you remember what motivated you to take part, or attracted you to this study in the first place?
2. In the beginning, how did you feel about the way the data was collected?
   1. *Did you have any concerns? Probe for feelings of surveillance or support.*
   2. *How did you feel about wearing a Fitbit all the time?*
   3. *How did you feel about the long-term commitment?*
3. Once you had started the RADAR study, how did you find wearing the Fitbit and using the apps?
   1. *Probe for aspects experienced as positive, interesting, helpful or unpleasant, difficult.*
   2. *Did your feelings change over the 12-month study period? If so, how?*
4. Did the notifications to answer the questionnaires affect how you went about your day-to-day life?
   1. *Did you find them intrusive/bothering at some point? Did they influence your mood?*
   2. *Did you always have time to complete them?*

**Devices’ impact on ED-relevant thoughts, feelings and behaviours**

I will now move on to questions about exercising and physical activity.

1. Once you had started using the Fitbit and the questionnaire apps, did your feelings about your exercising or activity change? If so, how?
   1. *Probe for attitude and awareness, both for better and worse.*
   2. *Prompts: Think about the beginning, middle and later stages of the study.*
2. What about your actual levels of physical activity, did they change as a result of using the devices? Please describe.
3. *Probe for helpful and less helpful effects.*
4. *Were there any specific features that you think may have led to these changes? If so, how?*

Now, I would like you to think about your eating.

1. Did using the devices influence your decisions and behaviours around food and eating in any way? Please describe.
2. *Probe for change in decision on what, how often and how much they ate.*
3. *Were there any specific features of the devices or the apps that you think may have led to you changing your eating habits? If so, how?*
4. *[If participant indicated a change in their eating due to the devices]: Did using the devices influence whether you felt you wanted to compensate, for example for eating too much or the ‘wrong’ foods?*

Next, I will ask you some questions about your thoughts related to your body while using the devices in the RADAR study.

1. Did the way you feel about your weight, or your body shape change as a result of using any of the devices or the apps? Please describe.
2. Did using the devices influence how conscious you were of your body? Please describe.
3. *Probe for helpful, reassuring, empowering, or uncomfortable, worried, stressed.*

**Feedback on the devices & closing questions**

The next questions will be about the devices themselves and their features.

1. You previously mentioned, that [insert previously mentioned feature] was stressful for you. How would you feel about being able to turn that feature off permanently?
   1. *If we talked about potentially unhelpful features at the beginning of the study and asked you to not use them, would you feel able to stick to that? Would you feel inclined to turn them back on?*
2. Based on your experience in the RADAR study, was there anything that you particularly valued about taking part?
3. While you participated in the study, or thinking back now: Is there an additional function that you would have found helpful for using the devices?
   1. *Probe for features related to app design, communication, support, etc.*
4. Have you used similar types of technology before participating in the RADAR-MDD study?
5. *Did you choose to continue to use the Fitbit after the study ended? If yes / If no, why?*
6. I have one final question. If a friend of yours who has an eating disorder was contemplating taking part in our future research study, what would you say to them?
   1. *Based on your own experience, what would you consider important to know before deciding whether to participate?*

***Stop recording***

*Thank participant, remind them of confidentiality and payment, confirm they have the researcher’s contact details*.
