# Supplementary material for: Perspectives on Remote Monitoring via Smartphones and Wearables Among Individuals With Lived Experience or at Risk of Eating Disorders (“This Could Go Very, Very Wrong”): Qualitative Interview Study
Source: JMIR Form Res. 2026 Jul 13;10:e86382. doi: 10.2196/86382 (PMC13362873; doi:10.2196/86382)
Supplement: Multimedia Appendix 3 [file formative-v10-e86382-s003.docx]

| **NAME** | | **DESCRIPTION** | **Frequency** | |
| --- | --- | --- | --- | --- |
| **Theme 1 – Remote measurement technology (RMT) as a research tool** | | | | |
| ***Participants’ motivation to participate in an RMT study and initial feelings*** | | | | |
| Personal relevance or experience | | Participant were curious about the RMT’s impact on their personal condition(s); sharing personal experiences to benefit future generations | 11 | |
| Excitement | | Excitement about taking part in the study | 2 | |
| Fitbit as an incentive | | The prospect of receiving a Fitbit served as motivator to take part | 7 | |
| Low intrusiveness | | The convenience of taking part from afar and the low effort required to take part appealed to participants | 14 | |
| Discreet data collection | | Being able to take part without having to explain to others |  | |
| Novelty | | The novelty of the concept of using personal digital devices in research | 11 | |
| Sense of belonging | | Feelings of importance, relevance, being part of a big research study / participant group | 7 | |
| Concerns about ED triggers | | History with eating disorders gave cause for concerns of some features of the Fitbit | 9 | |
| Scepticism / Privacy concerns | | (Initial) Hesitation about data tracking personal information sharing; desire for managing privacy settings | 4 | |
| ***Usability and practicality: Barriers and facilitators*** | | |  | |
| Ease of use | | Straight-forwardness and low cognitive effort required to use smartphone apps/wear a fitness device | 12 | |
| Familiarity with technology | |  |  | |
| Pre-existing digital literacy | | Participants had prior experience with the devices used / were comfortable using their phones (tech-savvy) | 7 | |
| Wearing a wrist device | | Unfamiliarity with wearable fitness trackers, concerns about discomfort; discomfort / annoyance skin reactions to wristband | 6 | |
| Required phone switch | | Unsure about switching to new mobile operating system | 2 | |
| Practical & logistical challenges | |  |  | |
| Scheduling / time constraints | | Intrusiveness and inconvenience of app notifications, added pressure on top of daily responsibilities | 11 | |
| Confusion about tasks | | Unclear when tasks would appear and how long they remained open | 3 | |
| Technical difficulties | | Issues with smartphone apps and Fitbit | 5 | |
| Reminders & routine | |  |  | |
| Adjustment and habit formation | | Adapting to wearing a Fitbit, wearing it became a habit | 17 | |
| Completion flexibility | | Adapting questionnaire scheduling to personal life: Completing questionnaires when able; delay completion until later that day | 9 | |
| Establishing routine | | Actively integrating reminders and questionnaire completion into daily life | 9 | |
| Importance of reminders | | Appreciation of prompts from app as reminder to complete tasks | 7 | |
| ***Emotional and cognitive engagement with active measures*** *(i.e. completing the tasks and questionnaires)* | | |  | |
| Gamification (of tasks) | | Enjoying the interactive ‘games’ aspect of the study; motivation to improve scores and continue participation | 11 | |
| Reflective awareness; supportive check-ins | | The opportunity for reflective moments and mood monitoring facilitated by answering the questionnaires; using questionnaires as a tool for self-improvement (e.g., family, friendships) | 16 | |
| Expectation & commitment | | Pragmatic approach to research where questionnaires are a to be expected part; Dedication to research, motivation to complete questionnaires properly once signed up | 17 | |
| Non-judgemental approach | | Age-appropriate, non-judgemental tone; appreciation of being taken seriously and not reduced to a diagnosis | 2 | |
| Ambiguity of mood patterns | | Nuance of mood fluctuations not reflected in questionnaires; lack of contrast between intense mood swings now vs. younger years | 3 | |
| Emotional burden of constant reporting | | Psychological strain of self-reporting data, including internal conflicts relating to data accuracy (sugarcoating emotions; not giving the “right data”, feeling “too good”), overwhelm from constant mood reflection | 15 | |
| Emotional investment in task completion | | Guilt / disappointment in oneself when unable to complete all questionnaires / pride and feelings of accomplishment when completing all | 6 | |
| Repetitiveness | | Repetitive questions across timepoints, leading to reduced engagement or questioning the value of repeated input | 2 | |
| ***Perception of being monitored*** | | |  | |
| Comfort with data collection | | Nonchalance about the fact of being monitored | 19 | |
| Trust in researchers and anonymity | | Clear communication and subsequent trust in the researchers that any data was anonymised | 3 | |
| Accountability through monitoring | | Being monitored serves as a motivational tool to check in with themselves / change behaviours | 3 | |
| Concerns about emotional transparency | | Fear that sensitive emotions can be picked up within provided data | 2 | |
| Supportive presence of tracking | | Personification of questionnaires; monitoring (esp. self-reporting) experienced as a subtle, companion-like presence that offers reassurance and emotional support; form of emotional check-in | 3 | |
| Observed adherence | | Sense of being observed, perceived awareness of researcher oversight / feeling watched or judged based on adherence | 2 | |
| The human link | |  |  | |
| Researcher-participant bridge | | Appreciation of the communication, support and feedback from the research team as a bridge between participants and the technology | 13 | |
| Isolating experience | | Lack of connection with others; solitary nature of an RMT study | 2 | |
| Age-related differences | | Age-related differences in technology adaptation; Balanced approach to being monitored self-attributed to age and logical reasoning vs. privacy concerns and more selective technology use of older generation | 5 | |
| **Theme 2 - Newfound knowledge. Health insights gained through Fitbit as a source of empowerment or stress** | | | | |
| ***Gaining health-related insights*** | | | |  |
| Health data enthusiasm | Fascination with general health (ie. non-activity) data collected via Fitbit | | | 11 |
| Value of data visualisation | Appreciation of being able to monitor progress using graphs | | | 5 |
| Awareness of activity data | Increased awareness of activity-related data (eg. daily steps, active minutes, calories burnt) | | | 18 |
| ***Emotional response to activity-related data*** | | | |  |
| Neutral attitude | Balanced view; interest in or curiosity about data | | | 3 |
| Empowerment/ sense of control | Empowered by knowledge; Ability to change one’s behaviour as response to gained awareness through data | | | 6 |
| Feelings of failure | Feeling frustrated about unachievable goals and feeling inadequate when not reached | | | 7 |
| Obsession with data | Unhealthy, obsessive relationship with data | | | 11 |
| Pressure to reach targets | Self-imposed or perceived stress from Fitbit to reach pre-set, standardised activity targets (desire for individualised targets) | | | 4 |
| Sense of achievement / Positive reinforcements | Feeling accomplished and proud of achieved (activity) goals; Celebrations of achieving milestones, reminders to move / keep going, visual rewards | | | 12 |
| ***Impact on self-perception*** | | | |  |
| Existing body image concerns | Concerns about one’s appearance existed before, regardless of receiving the Fitbit/accessing the data | | | 4 |
| Improved self-perception | General self-care leading to positive body image; External validation through visibility of Fitbit as indication of healthy intentions to others | | | 3 |
| Increased self-consciousness | Thinking more about one’s appearance and health | | | 3 |
| No change in body image | Perceived body image remained unchanged as result of having access to activity data | | | 3 |
| Stress | Being uncomfortable and stressed around body as result of Fitbit functions (eg. weight gain) | | | 2 |
| **Theme 3 - Behaviour modification. Increased awareness leading to behavioural change** | | | | |
| ***Influence on exercise choices*** | | | |  |
| Existing activity level | No change in routine | | | 4 |
| Increased exercising | Access to fitness-related data from Fitbit leads to increased exercising in terms of frequency and intensity | | | 19 |
| Influence on exercise choices | The types of exercising changed in response to seeing Fitbit data | | | 3 |
| Gamification element | Self-competition; Challenges of fitness targets, goal-driven exercising / outperforming oneself | | | 11 |
| General health focus as trigger | Health focus caused by seeing Fitbit data led to general health-promoting behaviours, incl. eating healthier and being more active | | | 3 |
| Exercise as a compensatory behaviour | Change / increase in exercising as result of seeing calorie data | | | 3 |
| ***Influence on eating choices*** | | | |  |
| Food as reward and punishment | Deserving of a treat / fasting depending on calories burnt | | | 4 |
| Healthier food choices | Eating healthier as result of Fitbit | | | 0 |
| Influence on portion size | Amount of food eaten changed as result of Fitbit (usually eating less) | | | 0 |
| No impact | Access to Fitbit data caused no change in eating behaviours | | | 5 |
| Restriction | Intentionally skipping meals | | | 2 |
| ***Wellbeing management*** | Self-awareness applied to managing wellbeing through Fitbit data, eg. calming down from panic attacks | | | 6 |
| **Theme 4 - Interplay with mood, engagement with the technology and interpretation of the data** | | | | |
| ***Fluctuating engagement with data*** | | | |  |
| Avoidance during low moods | Intentionally avoiding engaging with the technology and accessing the Fitbit data to prevent negativity | | | 5 |
| Casual curiosity | Ebb and flow in engagement with technology and obsession with tracking progress | | | 2 |
| Complex emotional journey | Initial discomfort with Fitbit (data), followed by shift towards self-acceptance and appreciation of improved well-being | | | 2 |
| Consistency | No change in how data was used / perceived throughout participation period | | | 2 |
| Increased engagement during positive moods | Active engagement with Fitbit during periods of high mood | | | 5 |
| Shifting expectations | Disillusionment and diminished expectations after initial hopes that Fitbit would help with participant’s well-being | | | 1 |
| ***Interpretation of data*** |  | | |  |
| Mood-dependent interpretation | Data progression is interpreted in light of current emotion | | | 2 |
| Amplifying existing emotions | Seeing data and Fitbit functions (eg. progression, reminders to move) amplify existing negative or positive moods | | | 10 |
| **Theme 5 - Health data as double edge sword. Using insights gained through recovery as coping strategy** | | | | |
| ***Recognising the risk*** | | | |  |
| Caution against fitness trackers | Participant advises against the use of fitness trackers for ED / at-risk individuals | | | 4 |
| Risk of triggering harmful behaviours | Participant is aware and acknowledges the potential danger of the wearable to trigger harmful ED-like behaviours | | | 5 |
| Self-deceptive nature of EDs | Reflections on younger self and acknowledging the inclination to use harmful features despite knowing its potential risk | | | 5 |
| ***Shifting perspectives / The recovery perspective: Reflections on the past and self insights gained through ED recovery*** | | | |  |
| Awareness of external influences | Gained ability to see how social media, peer pressure, cultural influences impact oneself | | | 2 |
| Grounded self-perception | Less inclined to rely on external validation for self-perception, being aware of one’s looks | | | 2 |
| Self-awareness about ED tendencies | Participant knows of their own obsessive or competitive tendencies | | | 13 |
| The power of knowledge | Gained understanding of (personal) nutritional needs caused shift in eating habits | | | 4 |
| ***Self-insights applied to data engagement*** | | | |  |
| Recognising the data’s value | Being able to see how data can be used in helpful ways / to support recovery | | | 2 |
| Self-regulation through feature avoidance | Intentionally avoiding features known to be harmful; self-restraint | | | 13 |
| ***Data applied to ED management*** | | | |  |
| Fitbit as a therapeutic tool | Fitbit as non-judgmental friend on your wrist vs the lack of human interaction | | | 11 |
| Fitbit’s value for recovery | Constructive impact of integrating technology into ED recovery; value in using the device (eg. recovery challenge, making conscious choices for healthier behaviours, life-saving potential) | | | 3 |
| Integration with therapy | Self-evaluation; applying things learned in therapy and Fitbit data together | | | 2 |
| Positive reinforcement for recovery efforts | Rewards for doing well and gentle reminders to do a little better; reassurance from seeing progress as sign of improved health | | | 3 |
| Supported self-monitoring | Intentionally changing behaviours to support recovery (eg. limit compulsive exercising, reminders to eat) | | | 7 |

| **NAME** | **DESCRIPTION** | | **Frequency** | |
| --- | --- | --- | --- | --- |
| **Theme 6 – Ethical implementation of RMT in EDs** | | | | |
| ***Advice for prospective study participants with EDs*** | | | |  |
| Awareness of personal limitations | | Identifying and acknowledging what might be harmful to oneself | | 4 |
| Encouragement to participate | | Enthusiasm about taking part, or at least trying/giving it a go; emphasising the benefits to oneself and others | | 11 |
| External consultation | | Involving friends/family/GP in making decision on participation | | 4 |
| Informed decision making | | Importance of gathering information and self-reflecting before deciding whether to take part | | 3 |
| Recommendation with caution | | Conditional recommendation to take part; advising to be careful/mindful | | 4 |
| ***Considerations for future RMT with young ED participants*** | | | |  |
| Balancing safety and autonomy | | Finding a balance between obscuring data for participants (not putting burden on them) vs. valuing participants’ autonomy (not patronising); Managing data access in a way that accommodates personal preferences and wellbeing while maintaining some control over the device’s features | | 6 |
| Importance of support resources | | Need for accessible support and communication options for participants | | 6 |
| Personalisation | | Transparency regarding the study’s aims and the significance of their role | | 2 |
| Community features | | Preference for (anonymous and optional) group challenges and engagement for an increased sense of belonging/less isolating | | 1 |
| Reassurance about researchers’ neutrality | | Keeping in mind embarrassment/shame/competitiveness around EDs | | 7 |
| Reassurance about voluntary nature of participation | | Making clear that participant can withdraw at any time without consequences, if the study isn’t right for them | | 2 |
| Remote blinding | | Importance of limiting participants' access to certain data | | 3 |
| Transparency about data handling | | Clearly communicating what data is being collected, where saved, how anonymised etc | | 9 |
| Updated devices | | Desire for improved or upgraded device features, eg. in terms of design and functionality | | 2 |
| Impact of age on RMT use | | Generational differences; Reflections on younger selves, likely unable to live up to research’s expectations and resulting guilt | | 4 |
| Clarify expectations | | Importance of detailed information on participation requirements (eg. on level of support available, acceptability of taking breaks) | | 2 |
